# Exploring the Prevalence and Characteristics of Obstructive Sleep Apnea Among Idiopathic Pulmonary Fibrosis Patients: A Systematic Review and Meta-Analysis

**DOI:** 10.7759/cureus.54562

**Published:** 2024-02-20

**Authors:** Calvin R Wei, Illahay Jalali, Jovanpreet Singh, Aishwarya Nagaraj, Mohammedsefa A Dari, Martha Mekonen Gdey, Monika Bai, Sujith K Palleti

**Affiliations:** 1 Research and Development, Shing Huei Group, Taipei, TWN; 2 Medicine, Tehran University of Medical Sciences, Tehran, IRN; 3 Internal Medicine, Adesh Institute of Medical Sciences and Research, Bathinda, IND; 4 Surgery, Pharmacology, Our Lady of Fatima University, Bangalore, IND; 5 Otolaryngology-Head and Neck Surgery, Addis Ababa University, Addis Ababa, ETH; 6 General Practice, Mekelle University, Mek'ele, ETH; 7 Obstetrics and Gynaecology, Shaikh Zaid Women Hospital, Larkana, PAK; 8 Nephrology, Louisiana State University Health Sciences Center, Shreveport, USA

**Keywords:** systematic review and meta-analysis, factors, prevalence, ideopathic pulmonary fibrosis, obstructive sleep apnoea

## Abstract

The aim of this meta-analysis was to scrutinize the prevalence, characteristics, and outcomes of obstructive sleep apnea (OSA) in individuals with ideopathic pulmonary fibrosis (IPF). We carried out this systematic review and meta-analysis in accordance with the guidelines outlined by the Preferred Reporting Items for Systematic Reviews and Meta-Analysis Statement (PRISMA). Two independent researchers systematically searched major databases, including MEDLINE/PubMed, EMBASE, and the Cochrane Library, from January 1, 2000, until December 31, 2023. We included all studies involving adult patients (age >18 years) with IPF that assessed the prevalence and characteristics of OSA in IPF patients. A total of seven studies involving a pooled sample of 411 patients were included in this meta-analysis. The pooled prevalence of OSA among individuals with IPF was found to be 70% (95% CI: 59 to 82%). Individuals with OSA exhibited a significantly higher mean body mass index (BMI) compared to their counterparts. While individuals with both IPF and OSA exhibited higher scores on the Epworth Sleepiness Scale (ESS) compared to those with IPF alone, the OSA group also showed lower oxygen saturation during sleep in comparison to non-OSA patients. In summary, OSA is a prevalent coexisting condition among individuals with IPF. This presence could worsen the nighttime oxygen saturation. Consequently, there is a need for more extensive studies involving more uniform participant groups.

## Introduction and background

Ideopathic pulmonary fibrosis (IPF) stands as the most prevalent form of chronic interstitial lung disease among elderly individuals [1]. The widely accepted theory elucidating the pathogenesis of this condition attributes it to recurrent micro-aggressions targeting the alveolar epithelium, induced by a variety of endogenous and exogenous factors such as viruses, cigarette smoke, gastroesophageal reflux, and pollutants [2]. This persistent alveolar assault results in a disruption of epithelial-mesenchymal interactions, culminating in aberrant repair processes for the injured epithelium [2].

Sleep breathing disorders (SBD), including obstructive sleep apnea (OSA) and sleep-related hypoxia, have gained recognition as significant features in IPF [3,4]. Notably, recent guidelines for the diagnosis and management of IPF highlight OSA as a crucial comorbidity [5]. Several studies have underscored the high prevalence of OSA syndrome in IPF patients, with up to 62% of newly diagnosed cases exhibiting moderate to severe OSA [6]. This suggests a potential temporal relationship where OSA may precede or coincide with the onset of IPF [7].

OSA, a prevalent chronic sleep-related breathing disorder, is characterized by recurrent episodes of apneas and hypopneas resulting from airway collapse or obstruction during sleep. These episodes lead to intermittent hypoxia, hypercapnia, arousals, and sleep fragmentation. Polysomnography (PSG) serves as the gold standard for OSA diagnosis, and continuous positive airway pressure (CPAP) application is the established treatment [8,9]. The clinical manifestations of OSA involve repetitive upper airway collapse-induced hypoxemia, hypercapnia, intrathoracic pressure swings, and sleep fragmentation, triggering arousal due to obstructive respiratory events [10]. Additionally, repetitive obstructive apnea, hypoxemia, and hypercapnia during sleep activate chemoreceptor reflexes, heightening sympathetic activity through interactions with baroreflex and pulmonary afferents, posing a risk for cardiovascular disease [11].

Recent investigations have revealed that IPF patients with OSA face an elevated risk of cognitive impairment, reduced sleep efficiency, diminished quality of life, and premature mortality compared to those without OSA [12,13]. However, the prevalence, predictive factors, and long-term impacts of OSA in IPF patients remain insufficiently clarified. Consequently, this study aims to scrutinize the prevalence, characteristics, and outcomes of OSA in individuals with IPF.

## Review

Methodology

We carried out this systematic review and meta-analysis in accordance with the guidelines outlined by the Preferred Reporting Items for Systematic Reviews and Meta-Analysis Statement (PRISMA).

Literature Search Strategy

Two independent researchers systematically searched major databases, including MEDLINE/PubMed, EMBASE, and the Cochrane Library, from January 1, 2000, until December 31, 2023. The keywords used for conducting the search included “idiopathic pulmonary fibrosis", “obstructive sleep insomnia," and "predictors,” along with their synonyms and medical subject heading (MeSH) terms. Additionally, reference lists of all included studies were also manually screened. We also explored additional data sources, including the reference lists of editorials, reviews in prominent medical journals, and databases containing gray, unpublished, or unprinted literature.

Study Selection

The inclusion criteria for studies were as follows: a) studies involving adult patients (age >18 years); b) studies exclusively focusing on patients with idiopathic pulmonary fibrosis (IPF); and c) studies assessing the prevalence and characteristics of OSA in IPF patients. Studies lacking original data were excluded, along with reviews, letters to the editor, comments, and conference abstracts. Studies including patients other than those with IPF were also excluded. Two review authors independently screened all titles and abstracts to assess the potential eligibility of studies, excluding those deemed clearly irrelevant. We identified studies and coded them as "retrieve" (eligible or potentially eligible/unclear) or "do not retrieve." Full-text study reports and publications were retrieved, and the review authors independently screened them to identify studies for inclusion while documenting reasons for excluding ineligible studies. Any disagreements were resolved through discussion, with attempts made to reach a consensus. If no agreement was reached, the principal author served as the arbiter.

Data Extraction and Quality Assessment

Two authors independently collected data, maintaining blindness to the authors and institutions of the studies included. Any discrepancies were resolved through a joint re-evaluation of the original article. The following details were extracted from each study: first author, publication year, region, study design, population characteristics, total population, number of patients who developed OSA, and characteristics of patients who developed OSA and those who did not.

The quality assessment of the included studies was conducted using the Newcastle-Ottawa scale, which considers criteria related to participant selection, comparability between groups, and outcome assessment. This scale aids in evaluating the quality of the evidence included in meta-analyses. Two authors performed the assessment independently, and any disagreements between them were resolved through discussion or by involving the principal investigator.

Statistical Analysis

We employed random-effect meta-analyses to determine the combined prevalence of obstructive sleep apnea (OSA), considering potential clinical and methodological variabilities in observational studies. Odds ratios (ORs) with 95% confidence intervals (CIs) were utilized for discontinuous parameters, while mean differences (MDs) with 95% CIs were chosen for continuous parameters. Significance was set at a p-value less than 0.05. Heterogeneity was assessed using a chi-squared test, and I2 with a 95% CI was used to quantify it. A p-value less than 0.05 was considered statistically significant. The analyses were conducted using Revman 5.4.1 software (The Cochrane Collaboration, Oxford, UK).

Results

**Figure 1:**
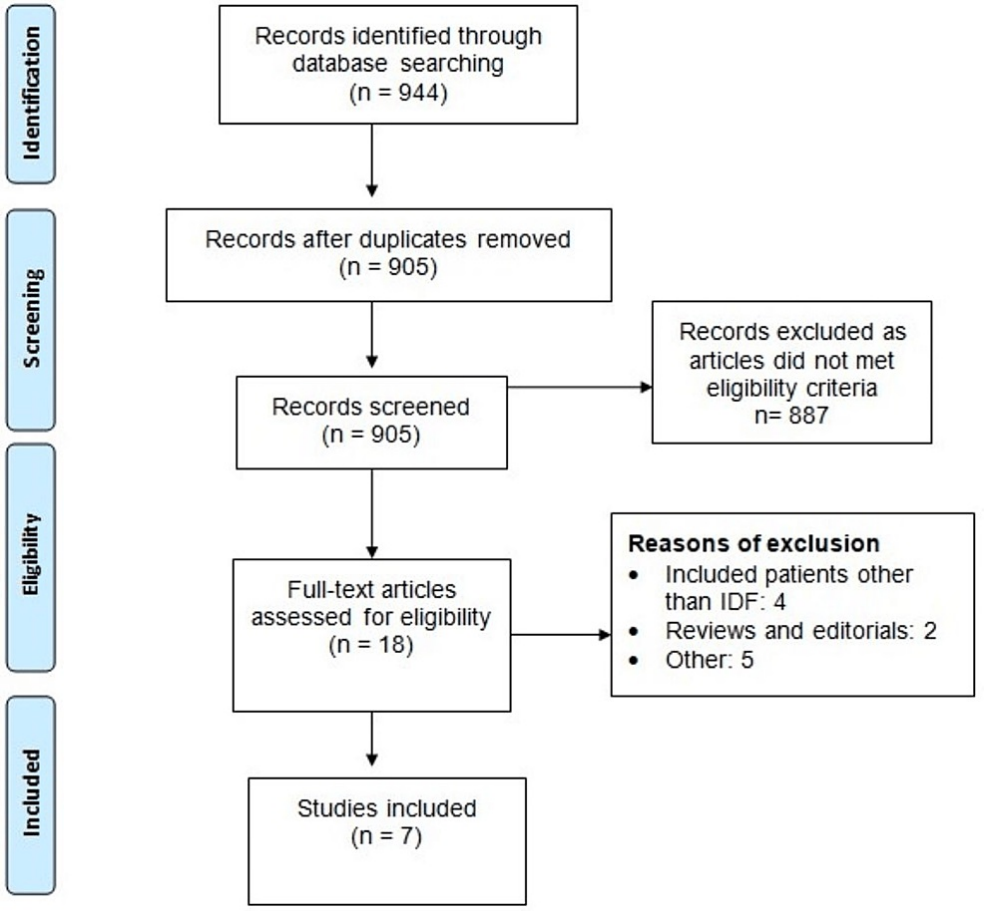
PRISMA flowchart of study selection

We initially identified 944 studies through online database searches. Following the elimination of duplicates, 905 studies underwent screening based on abstract or title. The full text of 18 studies was acquired, and a thorough evaluation was conducted according to pre-defined inclusion and exclusion criteria. Ultimately, seven studies [6-7,14-18] involving a total of 411 patients were included in this meta-analysis. Figure 1 provides a flow chart outlining the selection process and detailed identification. Table 1 presents the characteristics of the included studies. Among them, two studies were retrospective [15,17]. Geographically, two studies were conducted in Greece [16,17], while one study each took place in Korea [15], the United States [7], Argentina [18], France [16], and Italy [14]. The quality assessment of the included studies is detailed in Table 2.

**Table 1:** Characteristics of included studies OSA: Obstructive sleep apnea

Author	Year	Design	Region	Total	OSA (n)	Non-OSA (n)
Bosi et al. [14]	2019	Prospective	Italy	34	25	9
Gille et al. [6]	2017	Prospective	France	45	40	5
Lancaster et al. [7]	2009	Prospective	United States	50	44	6
Lee et al. [15]	2023	Retrospective	Korea	167	108	59
Mermigkis et al. [16]	2010	Prospective	Greece	34	20	14
Papadogiannis et al. [17]	2021	Prospective	Greece	45	29	16
Tabaj et al. [18]	2015	Retrospective	Argentina	36	17	19

**Table 2:** Quality assessment of included studies

Author	Selection	Comparison	Assessment	Overall
Bosi et al. [14]	2	2	2	Fair
Gille et al. [6]	3	1	3	Good
Lancaster et al. [7]	3	2	3	Good
Lee et al. [15]	3	2	2	Good
Mermigkis et al. [16]	3	2	3	Good
Papadogiannis et al. [17]	2	1	3	Fair
Tabaj et al. [18]	3	2	2	Good

Prevalence of OSA in IPF

The pooled prevalence of OSA among individuals with idiopathic pulmonary fibrosis (IPF) was found to be 70% (95% CI: 59 to 82%; I-square= 85.38%), with a range spanning from 47% to 89%. From the six studies that provided information on the severity of OSA encompassing a total of 258 OSA patients, it was revealed that mild OSA was present in 45.74% of the patients, while moderate to severe OSA was reported in 56.98% of the OSA patient population. We conducted a subgroup analysis based on the study design, revealing distinct prevalence rates for OSA. In prospective studies, the pooled prevalence of OSA was 76% (95% CI: 64 to 88%), showcasing a substantial occurrence. In contrast, retrospective studies yielded a pooled prevalence of 62% (95% CI: 55 to 68%).

Comparison of Characteristics Between Patients With OSA and Non-OSA

Table 3 presents a comparison of characteristics between individuals with obstructive sleep apnea (OSA) and those without OSA. No significant differences were observed between the two groups concerning age (p-value= 0.46) and gender (p-value = 0.41). The likelihood of having diabetes and gastroesophageal reflux disease (GERD) did not show significant variations between patients with OSA and those without OSA (p-value>0.05). However, individuals with OSA exhibited a significantly higher mean body mass index (BMI) compared to their counterparts.

**Table 3:** Comparison of characteristics between OSA and non-OSA OR: Odds ratio; CI: confidence interval: BMI: Body mass index; GERD: Gastroesophageal reflux disease; GAP: gender, age, and lung physiology ^ presented as the mean difference (95% confidence interval) * Significant at p-value<0.05

Variable	OR (95% CI)	P-value	I-square
Age (Years)^	1.19 (-1.97 to 4.36)	0.46	60%
Gender (Male)	1.24 (0.75 to 2.06)	0.41	0%
BMI^	2.11 (0.25 to 3.97)	0.03*	74%
Diabetes	1.68 (0.92 to 3.07)	0.09	0%
GERD	1.30 (0.66 to 2.56)	0.45	0%
GAP stage (III)	0.50 (0.20 to 1.22)	0.13	0%

OSA and Outcomes

Numerous studies have highlighted connections between obstructive sleep apnea (OSA) and various physiological and polysomnographic parameters, as detailed in Table 4. While individuals with both idiopathic pulmonary fibrosis (IPF) and OSA exhibited higher scores on the Epworth Sleepiness Scale (ESS) compared to those with IPF alone, the OSA group also showed lower oxygen saturation during sleep in comparison to non-OSA patients. However, the presence of OSA did not demonstrate a significant association with parameters such as six-minute walk distance (6MWD), forced vital capacity (FVC), total lung capacity (TLC), forced expiratory volume in one second (FEV1), diffusing capacity of the lungs for carbon monoxide (DLCO), as well as sleep stage durations (N1, N2, and N3).

**Table 4:** Comparison of outcomes between OSA and non-OSA MD: Mean difference; CI: Confidence interval; ESS: Epworth Sleepiness Scale; .6MWD: 6-minute walking distance; DLC: predicted diffusing capacity of the lung for carbon monoxide; FEV1: forced expiratory volume in 1 second, FVC: forced vital capacity; SpO2: resting room air pulse oximetry; TLC: total lung capacity. * Significant at p-value<0.05

Variable	MD (95% CI)	P-value	I-square
FVC (%)	1.56 (-2.04 to 5.16)	0.39	0%
FEV1 (%)	1.46 (-3.00 to 5.91)	0.52	0%
DLCO (%)	1.07 (-3.09 to 5.24)	0.61	0%
ESS	1.74 (0.69 to 2.79)*	0.001	0%
TLC (%)	-1.13 (-7.78 to 5.51)	0.74	0%
N1 (%)	0.10 (-0.34 to 0.54)	0.65	5%
N2 (%)	-2.04 (-4.94 to 0.87)	0.17	15%
N3 (%)	-0.39 (-0.86 to 0.08)	0.1	31%
6MWD (Meters)	-29 (-87.47 to 29.46)	0.33	38%
SpO2	-1.24 (-2.08 to -0.38)*	0.004	39%

Discussion

In this comprehensive meta-analysis, we have synthesized findings from seven clinical studies encompassing a total of 411 individuals with idiopathic pulmonary fibrosis (IPF). These studies contribute valuable insights into the prevalence and characteristics of obstructive sleep apnea (OSA) in IPF patients. The combined prevalence of OSA among individuals with IPF was determined to be 70% (95% CI: 59 to 82%). Notably, OSA stands out as one of the most prevalent sleep respiratory disorders, with estimates ranging from 6% to 17% in the general adult population [19]. Interestingly, our meta-analysis revealed that OSA is more prevalent among IPF patients compared to the general population.

In a prospective cross-sectional study involving 30 stable IPF patients, Sarkar et al. reported an overall OSA prevalence of 56.6%, with mild to moderate OSA being predominant in 76.5% of cases [13]. The intricate relationship between IPF and OSA presents a multifaceted challenge. It extends beyond molecular interactions, such as those involving the HIF-1α signaling pathway. Reports suggest that the diminished lung capacity observed in restrictive pulmonary diseases, such as IPF, may contribute to the destabilization of the upper airways, consequently leading to the development of OSA [20,21].

One notable risk factor significantly linked to obstructive sleep apnea (OSA) in individuals with idiopathic pulmonary fibrosis (IPF) is body mass index (BMI). Previous studies have consistently identified obesity or high BMI as prevalent risk factors for OSA in both the general population and IPF patients. The prevalence of OSA is nearly double in obese or severely obese individuals compared to those with normal weight [22,23]. The mechanism behind this association may involve fat deposition at specific sites, particularly in tissues surrounding the upper airway, leading to a reduced airway lumen and increased susceptibility to collapse, thus predisposing individuals to apnea [24]. However, the interplay between OSA and obesity is intricate; while evidence suggests that obesity, including visceral obesity, may contribute to OSA, recent studies propose that OSA itself could lead to weight gain [25-27].

Contrary to BMI, this meta-analysis did not identify any association between obstructive sleep apnea and pulmonary function tests (PFTs), including forced vital capacity (FVC), forced expiratory volume in one second (FEV1), and diffusing capacity of the lungs for carbon monoxide (DLCO). A potential explanation for this lack of correlation could be the mild reduction in lung volumes observed, consistent with early-stage IPF. OSA primarily involves temporary collapse or partial obstruction of the upper airway during sleep, causing breathing pauses and airflow disruptions. While PFTs evaluate lung volumes, capacities, and airflow, they may not directly capture the specific airway obstructions characteristic of OSA. Therefore, even if early-stage IPF is present with mild alterations in lung volumes, it may not necessarily reflect the severity of OSA [28].

The widely employed Berlin Questionnaire serves as a screening tool for identifying individuals at risk of obstructive sleep apnea (OSA). Developed to assess factors such as snoring, daytime sleepiness, high blood pressure, and body mass index (BMI), it aids in identifying those potentially susceptible to OSA [29]. In a study by Lee et al., the Berlin Questionnaire score was significantly higher in patients with OSA compared to those without, affirming its efficacy as a screening tool for predicting OSA. Given the practical challenges and resource implications associated with polysomnography, employing the Berlin Questionnaire as a preliminary screening tool for patients with idiopathic pulmonary fibrosis (IPF) could prove beneficial [30]. Nevertheless, further research is essential to validate the utility of the Berlin Questionnaire in diagnosing OSA in individuals with IPF.

Recent findings underscore the impact of nocturnal hypoxemia in OSA on the exacerbation of pulmonary hypertension in the course of IPF, leading to a worsened prognosis and increased mortality among IPF patients [31]. Intermittent hypoxemia, a characteristic manifestation of OSA, arises from airway obstruction and increased resistance. This intermittent hypoxemia triggers oxidative stress and inflammation, contributing to the development of OSA-related comorbidities [32]. The cumulative hypoxemia during sleep in IPF patients may adversely affect lung function by impairing the diffusing capacity of the lungs for carbon monoxide (DLco), inducing pulmonary vasoconstriction with ventilation/perfusion mismatch, and accelerating the progression of IPF [33]. Consequently, there is a compelling need to screen for OSA in patients with IPF, confirming the presence and severity of nocturnal hypoxemia and diagnosing OSA.

Our study is subject to several methodological limitations that warrant consideration when interpreting the findings. First, the majority of the included studies were conducted at single centers with small sample sizes, and certain clinical indicators were only assessed in a limited number of studies. For example, a few studies contributed to the analysis of important lung function parameters or comorbidities, resulting in a pooled analysis based on only three or four studies. This limitation may compromise the robustness of the meta-analysis and restrict the generalizability of the results. Small meta-analyses with limited studies may also introduce undetected heterogeneity, emphasizing the need for caution when interpreting pooled results from such subgroups. Future studies should incorporate appropriate multivariate analyses to explore the influence of obstructive sleep apnea (OSA) on idiopathic pulmonary fibrosis (IPF) outcomes independently, accounting for factors such as sex, age, disease severity, and treatment.

## Conclusions

In conclusion, our meta-analysis, comprising 411 idiopathic pulmonary fibrosis (IPF) patients from seven studies, reveals a significant prevalence of obstructive sleep apnea (OSA) in this population, reaching 70% (95% CI: 59 to 82%). Notably, OSA prevalence is higher in IPF patients compared to the general adult population. Subgroup analyses based on study design indicate varying prevalence rates, with prospective studies showing a substantial occurrence at 76%, while retrospective studies reveal a prevalence of 62%. The association of OSA with specific clinical characteristics underscores the need for comprehensive screening of IPF patients to improve outcomes.
